# Discrepancies Among Hospitals and Regions in the Provision of Low-Value Care

**DOI:** 10.34172/ijhpm.2024.7876

**Published:** 2024-04-20

**Authors:** Yu-Chen Kuo, Kuan-Chia Lin, Elise Chia-Hui Tan

**Affiliations:** ^1^Institute of Hospital and Health Care Administration, National Yang Ming Chiao Tung University, Taipei, Taiwan.; ^2^Community Medicine Research Center, Institute of Hospital and Health Care Administration, National Yang Ming Chiao Tung University, Taipei, Taiwan.; ^3^Department of Health Services Administration, College of Public Health, China Medical University, Taichung, Taiwan.

**Keywords:** Low-Value Care, Overuse, Utilization, Aisa

## Abstract

**Background:** Low-value care (LVC) is a critical issue in terms of patient safety and fiscal policy; however, little has been known in Asia. For the purpose of better understanding the extent of LVC on a national level, the utilization, costs, and associated characteristics of selected international recommendations were assessed in this study.

**Methods:** This retrospective cohort study used the National Health Insurance (NHI) claims data during 2013-2017 to evaluate the LVC utilization. Adult beneficiaries who enrolled in the NHI program and received at least one of the low-value services in hospitals were included. We measured seven procedures derived from the international recommendations at the hospital level, and a composite measure was created by summing the total utilization of selected services to determine the overall prevalence and corresponding cost. The generalized estimating equation (GEE) model was adopted to estimate the association.

**Results:** A total of 1 970 496 episodes of LVC was identified among 1 218 146 beneficiary-year observations and 2054 hospital-year observations. Overall, the utilization rate of the composite measure increased from 150.70 to 186.23 episodes per 10 000 beneficiaries with the growth in cost from US$ 5.40 to US$ 6.90 million. LVC utilization was proportional to the volume of outpatient visits and length of stay. Also, hospitals with a large volume of outpatient visits (adjusted odds ratio [aOR]: 95% CI, 2.10: 1.26 to 3.49 for Q2-Q3, 2.88: 1.45 to 5.75 for ≥Q3) and a higher proportion of older patients (aOR: 95% CI, 1.06: 1.02 to 1.11) were more likely to have high costs.

**Conclusion:** The utilization and corresponding cost of LVC appeared to increase annually despite the relatively lower prevalence compared to other countries. Multicomponent interventions such as recommendations, de-implementation policies and payment reforms are considered effective ways to reduce LVC. Repeated measurements would be needed to evaluate the effectiveness of interventions.

## Background

**Key Messages**

**Implications for policy makers**

- With the aim of improving the quality of care while simultaneously reducing overheads, regular measurements pertaining to the overuse of services in healthcare systems could be used by government officials to strategize.
- The overall utilization and corresponding cost of low-value care (LVC) was lower in Taiwan than in other western countries; nonetheless, most of these services appeared to be increasing over the five-year study period.
- Hospitals varied widely in the provision of LVC, and the utilization of such services was associated with the size of hospitals, age of patients, and comorbidity status.
- International recommendations adopted in this study could be applied in Asian countries, and prioritizing interventions based on the related impact characteristics are seen as practical approaches to reduce the burden of LVC.

**Implications for the public**

Low-value care (LVC) is a critical issue in terms of patient safety and fiscal policy, since it not only provides limited benefit and increased risks of harm to patients, but also induces a cascade of unnecessary cost. Such services could be driven by multiple factors, eg, patient preference, caregivers’ medical litigation concerns and fee-for-service payment systems. As care recipients, citizens are partly responsible for reducing the overuse; however, lack of public involvement has been one of the barriers. Our research applied international recommendations which could be identified using nationwide administrative data, and assessed the prevalence of LVC in an Asian setting. The findings could facilitate the development of evidence-based patient education and shared decision-making. It is believed that raising public awareness through the education campaigns such as Choosing Wisely is the first step to promote dialogue among patients, providers, and payers as to the necessity of medical interventions.

Low-value care (LVC) is commonly defined as tests, procedures, or treatments that provide little or no benefit and/or increased risks of harm to patients.^1-3^ It can induce a cascade of unnecessary care-related cost.^4-6^ In an effort to stem the financial burden imposed by such services, policy makers and experts have passed a number of initiatives, such as “Do not Do” (National Institute for Health and Care Excellence)^7^ and “Choosing Wisely” (American Board of Internal Medicine).^8^ Researchers have also created country-specific lists of examples of LVC.

 Most previous research on LVC utilization were conducted in the United States,^2,4,9-11^ Canada,^12-14^ Australia,^15-18^ and European countries.^19,20^ It has been reported that the prevalence of specific low-value services could range from 0.1% to 91.5%, depending on the locations,^21,22^ geographic regions,^23,24^ and payment systems.^25-30^ Researchers identified a number of measures that are associated with the utilization of LVC, including patient characteristics (eg, age, sex, ethnicity, socioeconomic status, and comorbidities)^21,24,31^ and physician characteristics (eg, seniority, specialty, and patient panel size).^13,23,32^ Note that factors related to the utilization of LVC in Asia have yet to be elucidated and country-specific lists of low-value interventions have not been developed for most Asian countries. We posited that the research performed in other countries could be used to lay the groundwork for future work in this area.

 In 1995, Taiwan implemented a single-payer mandatory National Health Insurance (NHI) program, now encompasses over 99% of 23 million residents and 93% of the hospitals and clinics.^33^ The NHI program offers comprehensive healthcare service, including outpatient visit, hospitalization, examinations, prescriptions, rehabilitation, and home care, with 30% of the contracted facilities being public. The NHI program is known for its high accessibility and affordability. However, despite its success, there are concerns about the ineffective gatekeeping of specialist services and the general quality of care.^34^ In the current study, we aimed to assess the situation of LVC in terms of utilization, cost, and trend over a five-year period. We also evaluated characteristics associated with the increased risk of LVC at the hospital and regional levels.

## Methods

###  Data Source and Study Design

 We adopted a non-interventional, retrospective cohort design to measure the prevalence of LVC services and corresponding costs. We used the National Health Insurance Research Database (NHIRD) during 2013-2017, which was obtained from the Health and Welfare Data Science Center, Ministry of Health and Welfare (NHIRD_MOHW).

 The administrative data from NHI program contains information related to enrollment, demographics, outpatient visits, admissions, procedures, prescriptions, and relevant costs. Note that this information is well suited to measuring healthcare utilization and cost trends over time. Regional data (eg, educational level, the number of low-income households, and the number of specialists) has been made available by the government^35,36^ and linked to NHIRD data for follow-up analyses.

 We identified all patients received at least one of the selected LVC services between 2013 and 2017 as the study population. Individuals were excluded from the study on the basis of age (<20 years old at the time of visit), incomplete enrollment data, or incomplete demographic information.

###  Measuring Low-Value Care

 In this study, we estimated the utilization of seven LVC services: the prostate specific antigen (PSA) test for men aged over 75 years old, repeated X-ray bone densitometry in short intervals, preoperative chest radiography, preoperative echocardiogram, preoperative pulmonary function tests (PFTs), preoperative stress tests, and screening for carotid artery disease in asymptomatic adults. These low-value procedures were selected from the American Board of Internal Medicine Foundation’s Choosing Wisely initiative,^8^ the US Preventive Services Task Force Grade “D” recommendations,^37^ the National Institute for Health and Care Excellence guidelines,^7^ Choosing Wisely Canada^38^ and the NPS MedicineWise’s Choosing Wisely Australia initiative.^39^ All of the measures have been shown to provide little or no benefit under specific or general scenarios. The seven selected services were identified using the International Classification of Diseases, Ninth and Tenth Revision, Clinical Modification codes and procedural billing codes. The number of episodes and relevant cost of LVC were estimated. Details pertaining to the coding systems are summarized in Table S1 (Supplementary file 1).

 Episodes were included in this study only if they were recorded as the principal procedures, thereby allowing the attribution of waste to unnecessary hospitalization or ambulatory visits. Restrictions pertaining to principal procedures were meant to exclude episodes that would still occur during the hospitalization despite not undergoing these procedures. We then estimated the number of episodes and corresponding medical costs associated with LVC at the population level and hospital level. We adopted the patient-indication measure for LVC prevalence,^40^ concentrating on the proportion of patients with a specific indication (either examination or treatment) who received LVC services.

###  Characteristics of Beneficiaries

 Enrolment records such as patient age, sex, and whether an individual belonged to low-income household when they received LVC were included. From outpatient visits and admissions data, principal and secondary diagnoses within one year prior to an event were used to calculate the combined comorbidity score^41^ of each individual.

###  Hospital and Regional Characteristics

 To determine whether hospital and regional factors were related to the utilization of LVC, we included the following characteristics in our models: accreditation level (medical center, regional hospital, or local hospital), ownership (public, private, or non-profit hospital), annual volume of outpatient visits, annual length of stays, seniority of physicians, proportion of male patients, mean age of patients, and combined comorbidity score of all patients per hospital. We also examined regional variables based on the serving area of hospitals, including the mean combined comorbidity score of residents, the ratio of specialists to primary care physicians, the proportion of residents who completed senior secondary education, the proportion of low-income households, and remoteness (including mountainous area, offshore island, and district with insufficient medical resources).

###  Statistical Analysis

 We measured the prevalence of LVC and the total corresponding medical cost on a yearly basis within the affected population on a nationwide level. We then aggregated this data at the hospital level for inference purposes. We also created a composite measure by summing the total utilization of selected services in order to determine the overall prevalence and corresponding cost of LVC. The cost of LVC was presented in US dollars, based on an exchange rate of 1:30 (New Taiwan dollars).

 Categorical and ordinal variables were presented as the number and the percentage of occurrences, while continuous variables were presented as mean and standard deviation (SD). The annual volume of outpatient department visits and the length of stays in hospitals were grouped by quartiles into four subcategories. All regional level variables of interest were split at the median to form high and low groups. Trend analyses on the utilization rate of LVC services, the number of affected beneficiaries, the number of episodes, and the corresponding costs were performed using the general linear model. The generalized estimating equation (GEE) model was used to determine whether variations observed at the hospital and regional level were associated with the utilization of LVC services (in terms of the number of episodes per 10 000 beneficiaries). Affected hospitals were classified into high- and low-cost groups based on the 75th percentile of corresponding costs. We also analyzed the relationship between characteristics of interest and high-cost group using GEE.

 Additional analyses which excluded two sex-specific LVC services (eg, PSA test for men aged over 75 years old and repeated X-ray bone densitometry in short intervals)^42^ were performed to examine the substantive associated factors. All analyses were performed using SAS, 9.4 version (SAS, Gray, North Carolina) with the level of statistical significance set at P<.05 based on two-tailed tests.

## Results

###  Characteristics of Beneficiaries, Hospitals, and Region

 Between January 1, 2013 to December 31, 2017, 914 191 beneficiaries (about 1.03% of all beneficiaries) received at least one of the selected LVC services, for a total of 1 218 146 beneficiary-year observations. We identified 493 hospitals that were providing LVC for a total of 2054 hospital-year observations. Table 1 presents the baseline characteristics at the beneficiary, hospital, and regional levels. The mean age of affected beneficiaries was 68.97 years (SD, 15.63), most of whom were male (65.83%). The majority of identified hospitals were local facilities (75.26%), and 52.54% of them were private. The mean proportion of male patients treated in the hospitals was 42.81% and the average experience of physicians was 15.16 years (SD, 4.54). Only 2.78% of the hospitals were located in mountainous areas, offshore islands, or districts with insufficient medical resources.

**Table 1:** Baseline Characteristics of Beneficiaries, Hospitals and Regions of the Selected Low-Value Care Services, 2013-2017

**Characteristics**	**No. (%)**
**Beneficiary Level**	
No. of beneficiary-year	1 218 146
Year	
2013	220 612 (18.11)
2014	231 380 (18.99)
2015	241 155 (19.80)
2016	253 543 (20.81)
2017	271 456 (22.29)
Age (y), mean (SD)	68.97 (15.63)
Female	416 292 (34.17)
Combined comorbidity score, mean (SD)	1.33 (2.09)
≥4	164 801 (13.53)
Low-income household	14 429 (1.18)
**Hospital Level**	
No. of hospital-years	2054
Year	
2013	408 (19.86)
2014	414 (20.16)
2015	411 (20.01)
2016	414 (20.16)
2017	407 (19.81)
Accreditation level	
Medical center	99 (4.82)
Regional hospital	409 (19.91)
Local hospital	1546 (75.27)
Ownership	
Public hospital	391 (19.03)
Private hospital	1079 (52.54)
Non-profit hospital	584 (28.43)
Volume of outpatient visits	
<Q1	513 (24.98)
Q1-Q2	514 (25.02)
Q2-Q3	513 (24.98)
≥Q3	514 (25.02)
Volume of length of stays	
<Q1	513 (24.98)
Q1-Q2	514 (25.02)
Q2-Q3	513 (24.98)
≥Q3	514 (25.02)
Physician seniority (y), mean (SD)	15.16 (4.54)
Patient age (y), mean (SD)	59.31 (6.53)
Proportion of male patients, mean (SD)	42.8 (10.39)
Combined comorbidity score of patients, mean (SD)	0.82 (0.43)
**Regional Level**	
Combined comorbidity score of residents	
Low	1020 (49.66)
High	1034 (50.34)
Ratio of specialists to primary care physicians	
Low	978 (47.61)
High	1076 (52.39)
Proportion of secondary education completion	
Low	1002 (48.78)
High	1052 (51.22)
Proportion of low-income households	
Low	1027 (50.00)
High	1027 (50.00)
Remoteness	
Yes	57 (2.78)
No	1997 (97.22)

Abbreviations: SD, standard deviation; Q1, the first quartile; Q2, the second quartile (median); Q3, the third quartile.

###  Extent and Trend of Low-Value Care

**Figure 1:**
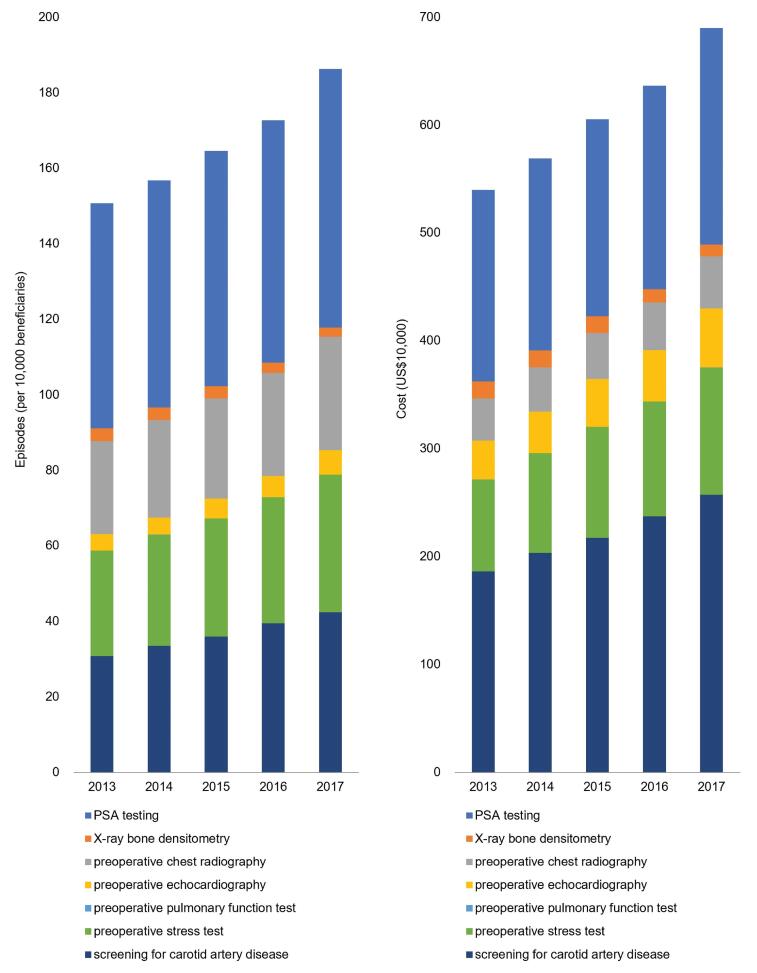
Utilization and Associated Cost of Selected Low-Value Care Services, 2013-2017. Counts of episodes refers to unique incidences of service provision, associated costs only include the fee for specific examinations within each episode of LVC services. Abbreviation: PSA, prostate specific antigen.

 In measuring the utilization of seven LVC services during the study period, we identified 1 970 496 distinctive episodes, with a corresponding cost of US$ 30.41 million. Figure 1 shows the utilization rate per 10 000 beneficiaries and the associated costs of the seven services. The most common low-value intervention was the PSA test for men aged over 75 years old, which increased from 59.63 per 10 000 beneficiaries in 2013 (US$ 1.77 million) to 68.46 per 10 000 beneficiaries in 2017 (US$ 2.01 million). The second most common intervention was screening for carotid artery disease in asymptomatic adults, which increased during the study period from 30.80 to 42.45 per 10 000 beneficiaries. The measure accounted for 36.14% of the total LVC services, increasing during the study period from US$ 1.86 to US$ 2.57 million (*P* for trend < .001). Table 2 demonstrates the trend on the utilization and costs of LVC. Other LVC services, such as preoperative chest radiography, preoperative echocardiography, and preoperative stress test, were also shown to increase in the prevalence and corresponding costs. Only the utilization of X-ray bone densitometry decreased in the prevalence and costs. As for the composite measure, the utilization rate increased from 150.70 to 186.23 episodes per 10 000 beneficiaries (ie, a 23.57% relative increase; *P* for trend = .001) with an increase in cost from US$ 5.40 to US$ 6.90 million (ie, a relative change of 27.78%; *P* for trend = .001).

**Table 2:** Utilization and Associated Cost of Selected and Composite Low-Value Care Services, 2013-2017

	**2013**	**2014**	**2015**	**2016**	**2017**	***P*** ** for Trend**
PSA testing						
Utilization rate of LVC, %	14.93	14.93	14.83	14.94	15.60	.202
No. of affected beneficiaries	89 326	90 905	92 359	94 794	100 445	.013
No. of episodes	139 912	142 033	147 881	152 941	163 490	.006
Associated cost, US$ 10 000	177.44	178.17	183.00	188.80	200.91	.017
X-ray bone densitometry						
Utilization rate of LVC, %	2.93	2.85	2.77	2.57	2.33	.007
No. of affected beneficiaries	7643	7503	7380	6054	5484	.021
No. of episodes	8036	7975	7769	6272	5669	.023
Associated cost, US$ 10 000	15.81	15.66	15.21	12.27	11.09	.021
Preoperative chest radiography						
Utilization rate of LVC, %	12.01	12.28	12.38	12.67	13.43	.018
No. of affected beneficiaries	49 110	51 650	53 686	55 601	61 270	.006
No. of episodes	57 613	60 890	63 044	65 181	71 816	.006
Associated cost, US$ 10 000	39.08	41.09	42.65	43.84	48.01	.006
Preoperative echocardiography						
Utilization rate of LVC, %	1.96	2.05	2.30	2.44	2.69	.001
No. of affected beneficiaries	8023	8609	9962	10 688	12 254	.001
No. of episodes	10 223	10 680	12 433	13 347	15 394	.003
Associated cost, US$ 10 000	36.15	38.33	44.46	47.58	36.15	.002
Preoperative PFT						
Utilization rate of LVC, %	0.0042	0.0067	0.0053	0.0062	0.0061	.330
No. of affected beneficiaries	17	28	23	27	28	.185
No. of episodes	17	30	24	28	30	.197
Associated cost, US$ 10 000	0.09	0.14	0.12	0.13	0.09	.081
Preoperative stress test						
Utilization rate of LVC, %	11.78	12.17	12.48	13.25	13.91	.002
No. of affected beneficiaries	48 163	51 168	54 148	58,132	63,463	.001
No. of episodes	65 521	69 510	74 388	79,349	86,946	.001
Associated cost, US$ 10 000	85.07	92.31	102.81	106.26	117.88	.001
Screening for carotid artery disease						
Utilization rate of LVC, %	37.02	37.74	38.43	39.86	40.45	.001
No. of affected beneficiaries	68 545	74 785	80 089	87,655	94,395	<.0001
No. of episodes	72 256	79 105	85 197	94,129	101,367	<.0001
Associated cost, US$ 10 000	186.19	203.48	217.29	237.45	257.26	.0001
Composite measure^a^						
No. of affected beneficiaries	220 612	231 380	241 155	253,543	271,456	.001
No. of episodes	353 578	370 223	390 736	411,247	444,712	.001
Associated cost, US$ 10 000	539.81	569.17	605.52	636.32	690.05	.001

Abbreviations: PSA, prostate specific antigen; LVC, low-value care; PFT, pulmonary function test. Note: The utilization rate of LVC indicates the proportion of patients with a specific indication receiving LVC services. ^a^ The composite measure was created by summing the total utilization and associated cost of selected LVC services.

###  Characteristics Associated With Low-Value Care Utilization

**Figure 2:**
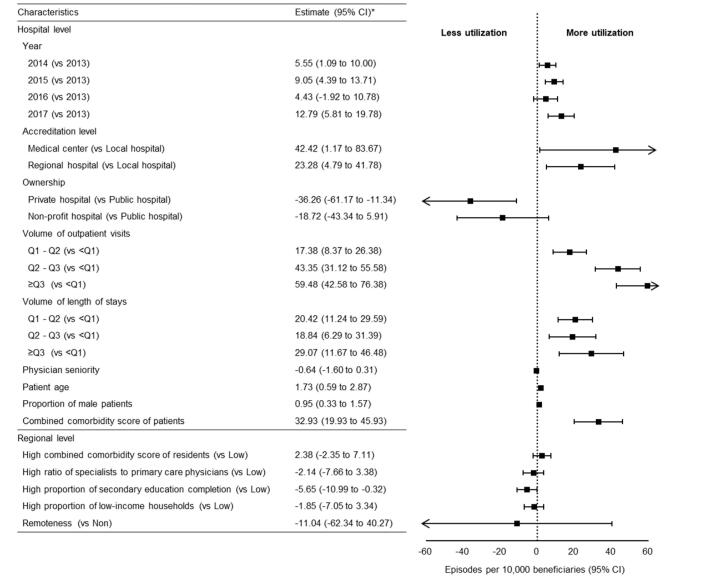
Correlation Between Utilization of Low-Value Care Services and Characteristics at the Hospital and Regional Levels. More utilization (on the right side) signifies that factors are associated with increased use of low-value care services, while less utilization (on the left side) indicates factors are correlated with reduced use of low-value service. * Estimates are the differences of LVC episodes per 10 000 beneficiaries from the reference group for each comparison group. Abbreviations: LVC, low-value care; CI, confidence interval.

**Figure 3:**
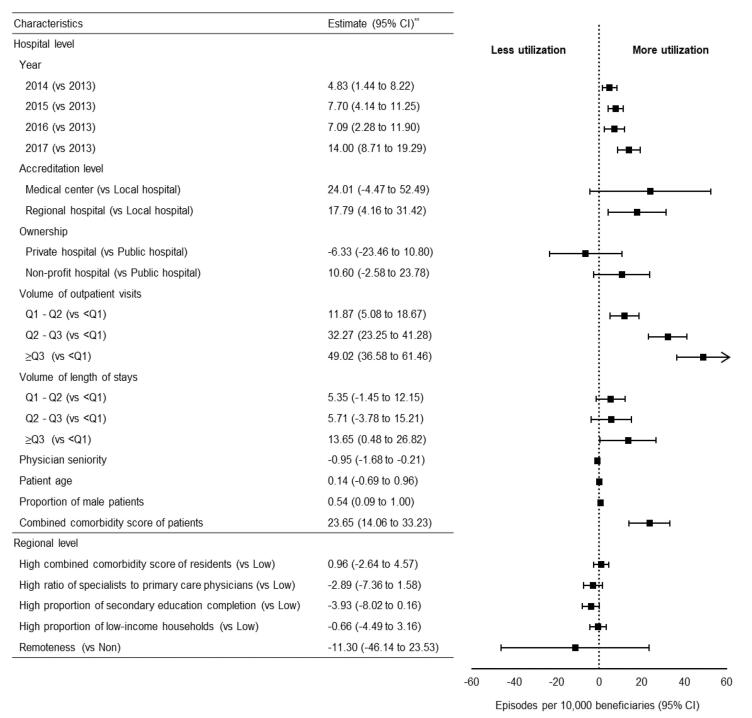
Correlation Between Utilization of Low-Value Care Services and Characteristics at the Hospital and Regional Levels, Excluding Sex-Specific Measures. Sex-specific LVC measures include PSA test and X-ray bone densitometry. Estimates are the differences of episodes per 10 000 beneficiaries from the reference group for each comparison group. More utilization (on the right side) signifies that factors are associated with increased use of LVC services, while less utilization (on the left side) indicates factors are correlated with reduced use of low-value service. Abbreviations: LVC, low-value care; PSA, prostate specific antigen; CI, confidence interval.

Figure 2 illustrates the association between characteristics of interest and the utilization of LVC services. In general, LVC appeared to increase over time; however, this relationship was not consistent in 2016 (episodes per 10 000 beneficiaries [95% CI], 5.55 [1.09 to 10.00] for 2014, 9.05 [4.39 to 13.71] for 2015, 4.43 [-1.92 to 10.78] for 2016, 12.79 [5.81 to 19.78] for 2017, respectively). Compared to local hospitals, medical centers (episodes per 10 000 beneficiaries [95% CI], 42.42 [1.17 to 83.67]) and regional hospitals (episodes per 10 000 beneficiaries [95% CI], 23.28 [4.79 to 41.78]) were more likely to provide LVC. Compared to public hospitals, private facilities were less likely to provide LVC (episodes per 10 000 beneficiaries [95% CI], -36.26 [-61.17 to -11.34]). LVC utilization of LVC was proportional to the annual volume of outpatient visits (episodes per 10 000 beneficiaries [95% CI], 59.48 [42.58 to 76.38] for ≥Q3, 43.35 [31.12 to 55.58] for Q2-Q3, 17.38 [8.37 to 26.38] for Q1-Q2, respectively) and length of stay (episodes per 10 000 beneficiaries [95% CI], 29.07 [11.67 to 46.48] for ≥Q3, 18.84 [6.29 to 31.39] for Q2-Q3, 20.42 [11.24 to 29.59] for Q1-Q2, respectively). The utilization was also positively correlated with the age of the patients, the proportion of male patients, and the presence of comorbidities. In terms of regional factors, LVC utilization was inversely proportional to the proportion of residents who completed senior secondary education (episodes per 10 000 beneficiaries [95% CI], -5.65 [-10.99 to -0.32]). Other characteristics were not significantly related to the utilization of LVC, including average combined comorbidity score, the ratio of specialists to primary care physicians, the proportion of low-income households and remoteness of location. Figure 3 presents the result of sensitivity analysis on the association between characteristics of interest and the utilization of non-sex-specific LVC services. We found that the correlation remained significantly positive between use and the proportion of male patients within hospitals (episodes per 10 000 beneficiaries [95% CI], 0.54 [0.09 to 1.00]).

###  Characteristics Associated With Costs of Low-Value Care

 Compared to low-cost facilities, high-cost ones were more likely to have a large number of outpatient visits and patient stays of longer duration (*P*< .0001) (Table S2, Supplementary file 1); they were also more likely to service older patients (mean [SD], 58.96 [6.85] vs 60.36 [5.34] years; *P*<.0001) and patients with multiple comorbidities (mean [SD], 0.80 [0.45] vs 0.88 [0.33]; *P*< .0001). Physicians in high-cost facilities tended to have less experiences than those in low-cost facilities (mean [SD], 15.52 [4.66] vs 14.09 [3.97] years; *P*< .0001). High-cost facilities tended to have a higher specialist to primary care physician ratio (50.49% vs 58.09%; *P*= .003) within a region where a higher proportion of the residents completed senior secondary education (48.86% vs 58.28%; *P*= .0002).

**Figure 4:**
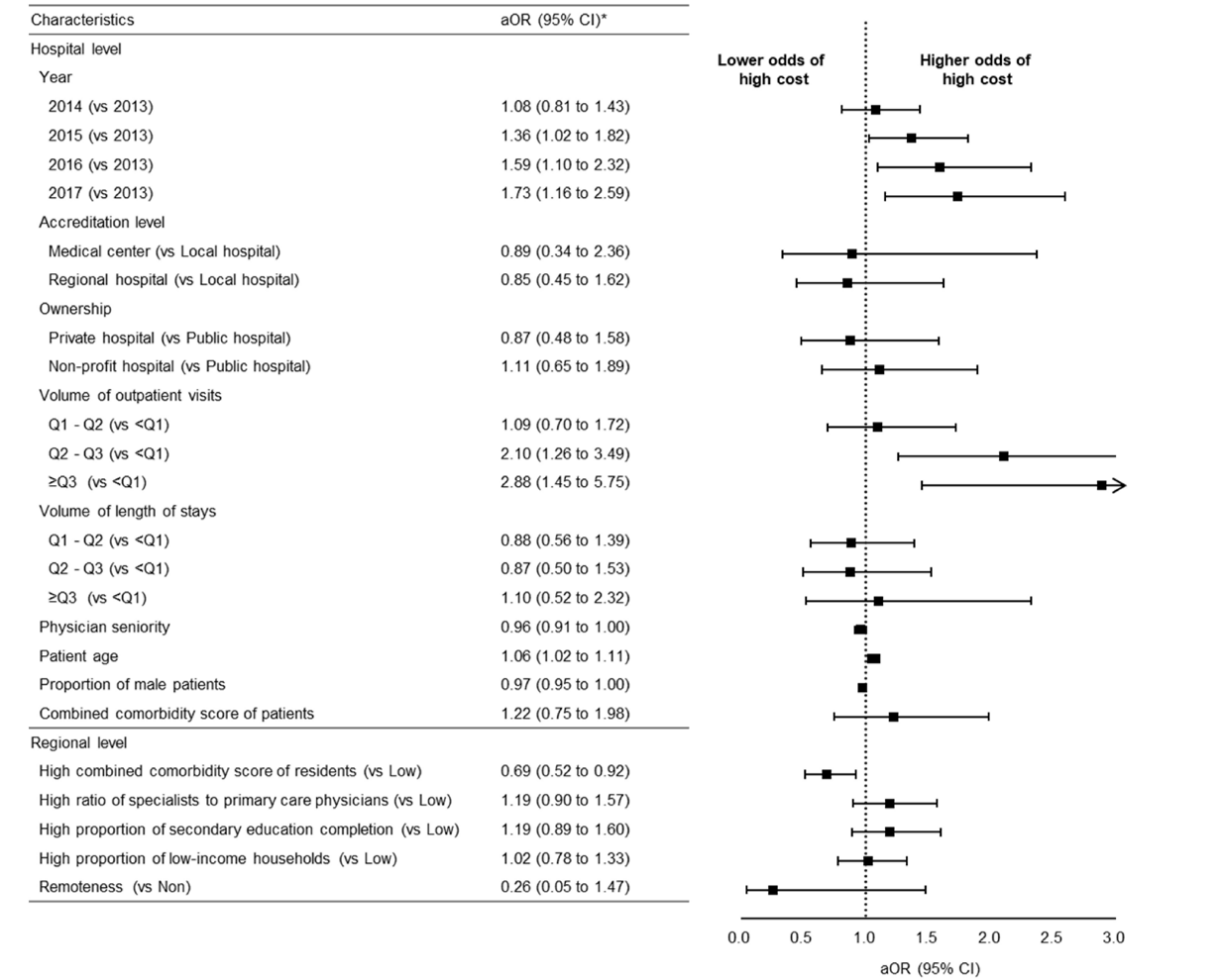
Correlation Between High Cost on Low-Value Care Services and Characteristics at the Hospital and Regional Levels. Higher odds of high cost (displayed on the right side of the figure) suggest that the factor is associated with an increased probability of higher LVC service cost, whereas lower odds of higher cost (shown on the left side) indicate a decreased probability of incurring higher costs for LVC services. Abbreviations: aOR, adjusted odds ratio; CI, confidence interval.

Figure 4 displays the relationship between characteristics of interest and associated costs of LVC. Costs were shown to increase yearly, with a corresponding increase in the adjusted odds ratio (aOR) from 1.36 (95% CI, 0.81 to 1.43) in 2015 to 1.73 (95% CI, 1.16 to 2.59) in 2017. A significantly positive correlation was observed between the volume of outpatient department visits and the cost associated with LVC. Hospitals with a larger volume of outpatient visits (aOR [95% CI], 2.10 [1.26 to 3.49] for Q2-Q3, 2.88 [1.45 to 5.75] for ≥Q3) and those treated a higher proportion of older patients (aOR [95% CI], 1.06 [1.02 to 1.11]) were more likely to be in the high-cost group. Hospitals with a higher proportion of male patients were less likely to be in the high-cost group (aOR [95% CI], 0.97 [0.95 to 1.00]). Regions with higher combined comorbidity scores were more likely to be in the low-cost group (aOR [95% CI], 0.69 [0.52 to 0.92]), meaning that areas with poor or fair health tend to have lower costs associated with LVC.

## Discussion

 LVC is a critical issue in terms of patient safety and fiscal policy.^18^ Most previous studies on the prevalence and utilization patterns of LVC were conducted in western countries. In the current study, we sought to extend their work to the situation at the hospital level. The results of this study demonstrate the extent of overuse, which also support the idea that the measurement of such services from several initiatives are applicable to an administrative database under an NHI program. Moreover, the comparison within and/or between hospitals provides preliminary information by which to formulate strategies to reduce costs. Our observations on utilization being associated with the volume of outpatient visits and the presence of multiple comorbidities indicates that future research should explore the causes of LVC and potential remedies. In addition, despite the abundance of low-value lists, there is still a limited understanding of the extent of LVC globally due to a lack of measurement, especially on Asia context. While historical measures of geographical variation in service utilization have provided insights into healthcare utilization patterns, they often do not account for the appropriateness of care.^40^ This study analyzed the nationwide patient-level data to evaluate the appropriateness of healthcare services based on patient characteristics and indications. Our results would enhance the understanding of LVC in an Asian setting.

 Researchers have highlighted utilization patterns and potential contributors to hospital-level LVC. The annual rate of LVC was 166.19 episodes per 10 000 beneficiaries during the five-year follow-up period, resulting in annual losses of US$ 6.08 million. The two most common low-value services were PSA tests for men aged over 75 years old and screening for carotid artery disease in asymptomatic adults. This should not be surprising, given the broad base of clinicians ordering these examinations.^43^ The findings corroborate their inclusion in Choosing Wisely lists and Do Not Do recommendations as targets for interventions.^44,45^ Note that PSA tests and preoperative chest radiography are low-cost (<US$ 50) yet commonly-used examinations. These results are consistent with prior research which determined that low-cost high-volume services contribute significantly to healthcare spending.^6,46^

 The observed increases in the utilization rate of LVC did not match previous observations indicating no change or a decrease in use.^11,30,47^ This can perhaps be attributed to the fact that Taiwan’s NHI provides easy access to healthcare with many beneficiaries engaging in doctor-shopping and undergoing overlapping examinations or treatments.^34,48^ According to the published statistics, the average number of visits per capita for ambulatory care was 13.2 in Taiwan in 2019, which was significantly higher than in Canada (6.6), Australia (7.3), and Germany (9.8).^49^ Earlier work has demonstrated that the Choosing Wisely Campaign and payment reforms would help reduce LVC^4,50^; nevertheless, little awareness has been raised among healthcare providers and policy makers across Asia.

 Our findings at the hospital level are consistent with previous studies. LVC utilization appears to be less of a problem in local hospitals, private hospitals, and the hospitals with fewer outpatient visits. Mafi et al^22^ formerly reported that community-based practices were less likely to promulgate LVC. We identified only a small number of regional factors that were predictive of LVC utilization or the associated costs. Badgery-Parker et al^15^ also reported that efforts to curb LVC should be at the hospital level rather than the regional level. Note that the factors most strongly correlated with LVC utilization were hospital service volumes and particularly ambulatory visits, indicating that larger institutions are more prone to unnecessary costs. These findings support preceding studies.^23,51^ Researchers have previously reported correlations between the utilization of LVC and male patients, old age, and multiple comorbidities.^21,23,24,47^ In the current study, we found that hospitals with older patient populations and greater comorbidity burden were more likely to provide LVC; moreover, the utilization were slightly higher in hospitals with a large proportion of male patients. It was very likely that sex-specific measures (eg, PSA tests and X-ray bone densitometry) could bias our results; therefore, we conducted sensitivity analyses to clarify these relationships. Overall, we determined that the correlation between sex and LVC remained significant.

 We believe that our study will contribute valuable insights into LVC within the Asian context. This study was subject to several limitations. First, the administrative claims data in this study lacked information related to clinical testing, which would have been valuable in defining low-value services more precisely. Note also that coding errors in large-scale databases may be inevitable. Nonetheless, we sought to minimize misclassification bias by applying procedural billing codes and adopting specific definitions available to facilitate the identification of LVC. Second, this study focused on a single country that provides unrestricted access to medical services under a universal coverage NHI program. As a result, our findings may not extrapolate to other healthcare systems, such as self-pay systems. The seven low-value services in this study are common among international recommendations and are easily defined in administrative data. Thus, our findings can be considered preliminary results relevant to the shaping of policies. Third, potential confounders at the physician level (eg, specialty and patient panel size) were not addressed in this study; however, we considered the seniority of physicians at the hospital level and the ratio of specialists to primary care physicians at the regional level as alternatives. We observed no correlation between these factors and LVC utilization.

## Conclusion

 This non-interventional, retrospective cohort study is considered a steppingstone to better understand the utilization of LVC and associated costs at the national level in an Asia setting. One approach to improving efficiency in healthcare resource allocation is the Choosing Wisely campaign, which promotes dialogue among payers, healthcare providers, and patients as to the necessity of medical interventions and examinations.^52^ Researchers have previously posited that multicomponent interventions, such as recommendations, disinvestment policies, and payment reforms, are the most effective approaches to reducing the utilization of LVC.^53-55^ Nonetheless, further studies will be needed to determine whether recommendations paired with policy changes, such as other bundled payment programs, would be able to reduce the burden of LVC. Repeating measurements will also be required to estimate the effectiveness of interventions over time.

## Acknowledgements

 We express our gratitude to the National Health Insurance Administration (NHIA) and the Health and Welfare Data Science Center for Ministry of Health and Welfare (HWDC_MOHW) for providing access to the databases utilized in this study.

## Ethical issues

 This study has been approved by the Joint Institutional Review Board (IRB no: 17-S-017-1).

## Competing interests

 Authors declare that they have no competing interests. Part of this study was presented as an oral presentation at the European Health Economic Association (EuHEA) Conference, Oslo, Norway, July 5-8, 2022. (Title of abstract: Characteristics of hospitals and areas associated with low-value care spending in the NHI Program, 2013-2017). Abstract of the conference presentation is available at https://euhea.eu/abstracts_conference_2022.html (Section: Efficiency Measurement in Health Care).

## Disclaimer

 All authors of this research paper have directly participated in the planning, execution, or analysis of the study. All authors of this paper have read and approved the final version submitted. It has not been published before and is not currently being considered for publication elsewhere. Nonetheless, the views expressed in this article do not reflect any official stance of the NHIA or MOHW. The authors had complete access to all data in the study and bear full responsibility for the integrity and accuracy of the data analysis.

## Funding

 This work was partly supported by grants from the National Science and Technology Council of Taiwan (grand no: NSTC 112-2410-H-039-005 -) and from the China Medical University (grand no: CMU112-N-14 and CMU112-MF-111). The funders did not play any role in the study’s design, the collection, analysis, or interpretation of data, the writing of the manuscript, or the decision to publish the results.

## Supplementary files

Supplementary file 1. Definition of Low-Value Care Services and Characteristics of Hospitals and Regions (Grouped According to the 75th Percentile of Corresponding Costs).
