# Supplementary material for: Discrepancies Among Hospitals and Regions in the Provision of Low-Value Care
Source: Int J Health Policy Manag. 2024 Apr 20;13:7876. doi: 10.34172/ijhpm.2024.7876 (PMC11270608; doi:10.34172/ijhpm.2024.7876)
Supplement: Supplementary file 1 — Definition of Low-Value Care Services and Characteristics of Hospitals and Regions (Grouped According to the 75th Percentile of Corresponding Costs). [file ijhpm-13-7876-s001.pdf]

Article title: Discrepancies Among Hospitals and Regions in the Provision of Low-Value Care

Journal name: International Journal of Health Policy and Management (IJHPM)

Authors' information: Yu-Chen Kuo[1], Kuan-Chia Lin[2], Elise Chia-Hui Tan[3]*

[1]Institute of Hospital and Health Care Administration, National Yang Ming Chiao Tung University, Taipei, Taiwan.

[2]Community Medicine Research Center, Institute of Hospital and Health Care Administration, National Yang Ming Chiao Tung University, Taipei, Taiwan.

[3]Department of Health Services Administration, College of Public Health, China Medical University, Taichung, Taiwan.

***Correspondence to:** Elise Chia-Hui Tan; Email: elisetam.g@gmail.com

Citation: Kuo YC, Lin KC, Tan ECH. Discrepancies among hospitals and regions in the provision of low-value care. Int J Health Policy Manag. 2024;13:7876. doi:10.34172/ijhpm.2024.7876

Supplementary file 1. Definition of Low-Value Care Services and Characteristics of Hospitals and Regions (Grouped According to the 75th Percentile of Corresponding Costs).

Table S1: Definition and Source of Selected Low-Value Care Services

Table S2: Characteristics of Hospitals and Regions With at Least 1 Low-Value Care Service, by the 75th Percentile of Associated Cost, 2013-2017

Table S1. Definition and Source of Selected Low-Value Care Services

Low-value care	Source	Operational definition
PSA test for men aged over 75 years old	CWA, USPSTF	Male patients aged over 75 years old and without history of prostate cancer received PSA test.
Repeated x-ray bone densitometry at frequent intervals	CWC, USPSTF	Patients with osteoporosis received X-ray bone densitometry in the current year and had received the same test at least once during the previous two years.
Preoperative chest x-ray	CWC, CWUS, NICE	Patients received chest x-ray within 30 days before a low- or intermediate-risk noncardiothoracic surgery.* Emergency cases were excluded.
Preoperative echocardiography	CWC, CWUS, NICE	Patients received echocardiography within 30 days before a low- or intermediate-risk noncardiothoracic surgery.* Emergency cases were excluded.
Preoperative pulmonary function test	NICE	Patients received pulmonary function test within 30 days before a low- or intermediate-risk noncardiothoracic surgery.* Emergency cases were excluded.
Preoperative stress test**	CWA, CWC, CWUS, NICE	Patients received stress test within 30 days before a low- or intermediate-risk noncardiothoracic surgery.* Emergency cases were excluded.
Screening for carotid artery disease in asymptomatic adults	CWA, CWUS, USPSTF	Patients without previous diagnosis of stroke, transient ischemic attack or focal neurological symptoms received common carotid artery test. Inpatients and emergency cases were excluded.

* Low- or intermediate-risk non-cardiothoracic surgeries contained breast procedures, colectomy, cholecystectomy, transurethral resection of the prostate, hysterectomy, orthopedic surgical procedures, corneal transplant, cataract removal, retinal detachment, hernia repair, lithotripsy, arthroscopy, and cholecystectomy.

** Stress tests included electrocardiography, echocardiography and nuclear medicine imaging.

Abbreviations: CWA, Choosing Wisely Australia; CWC, Choosing Wisely Canada; CWUS, Choosing Wisely Canada; CWUS, Choosing Wisely United States; NICE, National Institute for Health and Care Excellence; USPSTF, U.S. Preventive Services Task Force.

Table S2. Characteristics of Hospitals and Regions With at Least 1 Low-Value Care Service, by the 75th Percentile of Associated Cost, 2013-2017

	Low-cost group	High-cost group	
Characteristics	No. (%)	No. (%)	*P* value
Hospital level			
No. of hospital-years	1,541 (75.00)	513 (15.00)	
Year			.110
2013	322 (20.90)	86 (16.76)	
2014	320 (20.77)	94 (18.32)	
2015	302 (19.60)	109 (21.25)	
2016	304 (19.73)	110 (21.44)	
2017	293 (19.01)	114 (22.22)	
Accreditation level			<.0001
Medical center	57 (3.70)	42 (8.19)	
Regional hospital	254 (16.48)	155 (30.21)	
Local hospital	1,230 (79.82)	316 (61.60)	
Ownership			<.0001
Public hospital	269 (14.76)	122 (23.78)	
Private hospital	873 (56.65)	206 (40.16)	
Non-profit hospital	399 (25.89)	185 (36.06)	
Volume of outpatient visits			<.0001
<Q1	448 (29.07)	65 (12.67)	
Q1 - Q2	443 (28.75)	71 (13.38)	
Q2 - Q3	349 (22.65)	164 (31.97)	
≥Q3	301 (19.53)	213 (41.52)	
Volume of length of stays			<.0001
<Q1	427 (27.71)	86 (16.67)	
Q1 - Q2	420 (27.26)	94 (18.32)	

Q2 - Q3	365 (23.69)	148 (28.85)	
≥Q3	329 (21.35)	185 (41.52)	
Physician seniority, mean (SD), y	15.52 (4.66)	14.09 (3.97)	<.0001
Patient age, mean (SD), y	58.96 (6.85)	60.36 (5.34)	<.0001
Proportion of male patients, mean (SD), %	42.84 (10.93)	42.72 (8.58)	.788
Combined comorbidity score of patients, mean (SD)	0.80 (0.45)	0.88 (0.33)	<.0001
Regional level			
Combined comorbidity score of residents			.593
Low	760 (49.32)	260 (50.68)	
High	781 (50.68)	253 (49.32)	
Ratio of specialists to primary care physicians			.003
Low	763 (49.51)	215 (41.91)	
High	778 (50.49)	298 (58.09)	
Proportion of secondary education completion			.0002
Low	788 (51.14)	214 (41.72)	
High	753 (48.86)	299 (58.28)	
Proportion of low-income households			.799
Low	768 (49.84)	259 (50.49)	
High	773 (51.16)	254 (49.51)	
Remoteness			.002
Yes	53 (3.44)	4 (0.78)	
No	1,488 (96.56)	509 (99.22)	
